# Supplementary material for: Pseudomonas mRNA 2.0: Boosting Gene Expression Through Enhanced mRNA Stability and Translational Efficiency
Source: Front Bioeng Biotechnol. 2020 Jan 24;7:458. doi: 10.3389/fbioe.2019.00458 (PMC6993053; doi:10.3389/fbioe.2019.00458)
Supplement: Supplementary file 1 [file Table_1.docx]

Supplementary Material

Table S1. Primers used for cloning and qPCR. Lower-case letters represent nucleotide overhangs used for Gibson cloning. Upper-case letters represent nucleotides binding to the amplicon.

| Primer | Sequence | Description |
| --- | --- | --- |
| DN_39 | gcatggatgaactctacaaataATAGAGGGACAAACTCAAGGTC | Opt_Syn42_GFP backbone (fwd) |
| DN_40 | cctttatgaattcccatgaTCATTAGAAAACCTCCTTAGCATG | Opt_Syn42_GFP backbone (rev) |
| DN_41 | CATGCTAAGGAGGTTTTCTAATGATCATGGGAATTCATAAAGG | Opt_Syn42/Syn35_GFP insert (fwd) |
| DN_42 | GACCTTGAGTTTGTCCCTCTATTATTTGTAGAGTTCATCCATGC | Opt_Syn42/Syn35/SPA75_GFP insert (rev) |
| DN_55 | gaactctacaaataaAACGAGAAAAGCCAACCTGCGGGTTGG | Tra_Syn42 backbone (fwd) |
| DN_56 | cattagaaaacctcCTCCTAGGCGTGCAATTATACCTGGCCGC | Tra_Syn42 backbone (rev) |
| DN_57 | taattgcacgcctaggAGGAGGTTTTCTAATGATCATGGG | Tra_Syn42_GFP insert (fwd) |
| DN_58 | gttggcttttctcgttTTATTTGTAGAGTTCATCCATGCCG | Tra_Syn42_GFP insert (rev) |
| DN_247 | AACGAGAAAAGCCAACCTG | Tra_Syn35/SPA75_GFP; Tra_Syn42/Syn35/SPA75_mCherry backbone (fwd) |
| DN_248 | TAGAAAACCTCCTCCTAGGCG | Tra_Syn42_mCherry backbone (rev) |
| DN_270 | CCTAGGCCCCAAATTATAATTCTAAAC | Tra_Syn35_GFP; Tra_Syn35_mCherry backbone (rev) |
| DN_274 | CCTAGGCGTGCAATTATAGTATC | Tra_SPA75_GFP; Tra_SPA75_mCherry backbone (rev) |
| DN_249 | gcctaggaggaggttttctaATGGTGAGCAAGGGCGAG | Tra_Syn42_mCherry insert (fwd) |
| DN_250 | gcaggttggcttttctcgttTTACTTGTACAGCTCGTCCATG | Tra_Syn42/SPA75_mCherry insert (rev) |
| DN_271 | attataatttggggcctaggAGGAGGTTTTCTAATGATC | Tra_Syn35_GFP insert (fwd) |
| DN_272 | gcaggttggcttttctcgttTTATTTGTAGAGTTCATCCATG | Tra_Syn35/SPA75_GFP insert (rev) |
| DN_281 | attataatttggggcctaggAGGAGGTTTTCTAATGGTG | Tra_Syn35_mCherry insert (fwd) |
| DN_250 | gcaggttggcttttctcgttTTACTTGTACAGCTCGTCCATG | Tra_Syn35_mCherry insert (rev) |
| DN_275 | actataattgcacgcctaggAGGAGGTTTTCTAATGATC | Tra_SPA75_GFP insert (fwd) |
| DN_276 | actataattgcacgcctaggAGGAGGTTTTCTAATGGTG | Tra_SPA75_mCherry (fwd) |
| DN_229 | TAGAGGGACAAACTCAAG | Opt_Syn35/SPA75_GFP; Opt_Syn42/Syn35/SPA75_mCherry backbone (fwd) |
| DN_230 | TAGAAAACCTCCTTAGCATG | Opt_Syn35_GFP; Opt_Syn42/Syn35/SPA75_mCherry backbone (rev) |
| DN_267 | TTAATTAAGACGTCTTGACATAAGC | Opt_SPA75_GFP backbone (rev) |
| DN_245 | catgctaaggaggttttctaATGGTGAGCAAGGGCGAG | Opt_Syn42/Syn35/SPA75_mCherry insert (fwd) |
| DN_236 | accttgagtttgtccctctaTTACTTGTACAGCTCGTCCATG | Opt_Syn42/Syn35/SPA75_mCherry insert (rev) |
| DN_269 | tgtcaagacgtcttaattaaGCCCATTGACAACACTATTTTTTGATACTATAATTGCACGCCTAGGAGCTGTCACCGG | Opt_SPA75_GFP insert (fwd) |
| DN_127 | CGGCCGCGCTAGCACTGA | *nag*R_Tra/Opt_GFP/mCherry/ilvB_aldB backbone (fwd) |
| DN_288 | CCGACGTCGCATGCTCCT | *nag*R_Tra/Opt_GFP/mCherry/ilvB_aldB backbone (rev) |
| DN_289 | agaggagcatgcgacgtcggAGGAGGTTTTCTAATGATC | *nag*R_Tra_GFP insert (fwd) |
| DN_142 | ggtcagtgctagcgcggccgTTATTTGTAGAGTTCATCCATGC | *nag*R_Tra_GFP insert (rev) |
| DN_290 | agaggagcatgcgacgtcggAGCTGTCACCGGATGTGC | *nag*R_Opt_GFP/mCherry insert (fwd) |
| DN_291 | ggtcagtgctagcgcggccgAAGAAGGTCAATCATAAAGGCCAC | *nag*R_Opt_GFP/mCherry/*aldB* insert (rev) |
| DN_292 | agaggagcatgcgacgtcggAGGAGGTTTTCTAATGGTG | *nag*R_Tra_mCherry insert (fwd) |
| DN_293 | ggtcagtgctagcgcggccgTTACTTGTACAGCTCGTC | *nag*R_Tra_mCherry insert (rev) |
| DN_298 | agaggagcatgcgacgtcggACAGGAGACTTTCTAATGGCC | *nag*R_Tra_*ilvB* insert (fwd) |
| DN_299 | ctaggcgtgcTCACTCGCCGACCATCTC | *nag*R_Tra_*ilvB* insert (rev) |
| DN_300 | cggcgagtgaGCACGCCTAGGAGGAGGTTTTC | *nag*R_Tra_*aldB* insert (fwd) |
| DN_301 | ggtcagtgctagcgcggccgTCACTTGCGCTCGCTTTC | *nag*R_Tra_*aldB* insert (rev) |
| DN_302 | agaggagcatgcgacgtcggAGTCCGTAGTGGATGTGTATC | *nag*R_Opt_*ilvB* insert (fwd) |
| DN_303 | ctaggcgtgcAAAGTGATAATCATAAAGGCCAC | *nag*R_Opt_*ilvB* insert (rev) |
| DN_294 | ttatcactttGCACGCCTAGGAGCTGTC | *nag*R_Opt_*aldB* insert (fwd) |
| DN_537 | AGGAGGTTTGTATCTCTAATG | *att*Tn7_Tra_GFP backbone (fwd) |
| DN_534 | AGCTGTCACCGGATGTGC | *att*Tn7_Opt_GFP backbone (fwd) |
| DN_415 | TTGACAGCTTATCATCGATAAAC | *att*Tn7_Tra/Opt_GFP backbone (rev) |
| DN_532 | tatcgatgataagctgtcaaTTGACACCATCGAATGGTGC | *att*Tn7_Tra/Opt_GFP insert (fwd) |
| DN_538 | attagagatacaaacctcctGCGGCCTAGGGTGTGAAATTG | *att*Tn7_Tra_GFP insert (rev) |
| DN_539 | aagcacatccggtgacagctGCGGCCTAGGGTGTGAAATTG | *att*Tn7_Opt_GFP insert (rev) |
| DN_392 | CACCGCAGACAAACAGAAGA | qPCR primer msfGFP (fwd) (Otto et al., 2019) |
| DN_393 | ACTGGGTGGACAGGTAGTGG | qPCR primer msfGFP (rev) (Otto et al., 2019) |
| DN_549 | TTGGCCCAGAGGAAATCAC | qPCR primer rpoB (fwd) |
| DN_550 | GGCACCGACGTAGACAATAC | qPCR primer rpoB (fwd) |

*aldB, B. brevis*

CCATGGTACCACCGTCAAAAAAAACGGCGCTTTTTAGCGCCGTTTTTATTTTTCAACCTTCGCATACGCTACTTGCATTACAGTTTACGAACCGAACAGGCTTATGTCAAGACGTCTTAATTAAGCCCATTGACAAGGCTCTCGCGGCCAGGTATAATTGCACGCCTAGGAGCTGTCACCGGATGTGCTTTCCGGTCTGATGAGTCCGTGAGGACGAAACAGCCTCTACAAATTTTGTTTAAGCCCAAGTTCACTTAAAAAGGAGATCAACAATGAAAGCAATTTTCGTACTGAAACATCTTAATCATGCTAAGGAGGTTTTCTAATGAAGAAGAACATTATCACGTCGATTACCAGCTTGGCGTTGGTCGCGGGCCTCAGCTTGACCGCGTTCGCCGCAACGACCGCCACGGTGCCCGCCCCCCCGGCCAAGCAGGAAAGCAAGCCCGCCGTCGCCGCCAACCCGGCTCCTAAGAATGTGCTGTTCCAGTACAGCACCATCAACGCCCTTATGCTGGGCCAGTTCGAAGGCGACCTGACGTTGAAGGATCTGAAGTTGCGCGGCGATATGGGCCTGGGCACGATCAACGATCTTGACGGCGAAATGATCCAAATGGGCACCAAATTCTACCAAATCGACTCCACGGGCAAACTGAGCGAACTCCCAGAATCCGTAAAGACCCCATTCGCCGTCACGACCCACTTCGAGCCAAAGGAGAAGACGACCCTGACCAACGTGCAGGATTACAACCAGCTGACCAAGATGCTGGAGGAAAAATTCGAGAACAAAAACGTCTTCTACGCCGTAAAACTGACCGGGACCTTCAAGATGGTGAAGGCCCGCACCGTGCCGAAGCAAACCCGTCCATACCCACAACTGACCGAGGTGACGAAGAAGCAGAGCGAGTTCGAGTTCAAGAACGTGAAGGGTACGCTGATCGGCTTCTACACGCCGAACTACGCCGCCGCCCTGAATGTCCCCGGTTTCCATTTGCATTTCATCACGGAGGACAAAACGTCCGGCGGTCATGTACTGAACCTTCAGTTCGATAATGCGAACCTGGAGATCAGCCCCATCCACGAGTTCGACGTCCAGCTGCCCCATACCGATGATTTCGCCCATTCGGATTTGACCCAGGTGACCACGTCGCAAGTACATCAGGCCGAAAGCGAGCGCAAGTGATAGAGGGACAAACTCAAGGTCATTCGCAAGAGTGGCCTTTATGATTGACCTTCTTAACGAGAAAAGCCAACCTGCGGGTTGGCTTTTTTATGCAGCGGCCGC
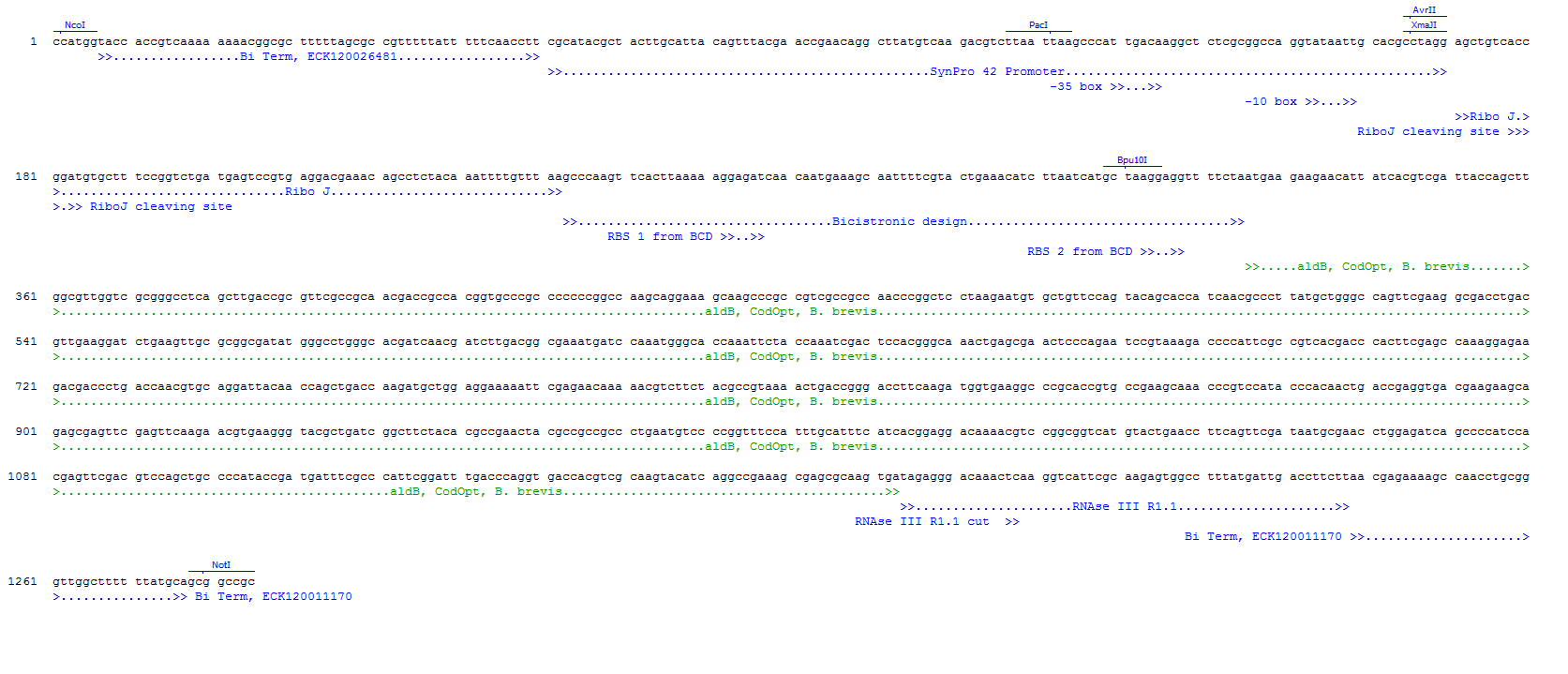
**Supplementary Figure 1.** Annotated sequence of the synthetic DNA fragment containing the bidirectional terminators, the synthetic SynPro42 promoter, RiboJ, BCD2, codon optimized *aldB* gene from *B. brevis* and RNase III site R1.1.

*ilvB* C83S*, E. coli* K12

CCATGGAACGAGAAAAGCCAACCTGCGGGTTGGCTTTTTTATGCACGCATACGCTACTTGCATTACAGTTTACGAACCGAACAGGCTTATGTCAAGACGTCTTAATTAATCTACTTGACATCCGACATTCGCGACTGTATAATAAGTTGACCTAGGGAGTCCGTAGTGGATGTGTATCCACTCTGATGAGTCCGAAAGGACGAAACGGACCTCTACAAATAATTTTGTTTAAGGGCCCAAGTTCACTTAAAAAGGAGATCAACAATGAAAGCAATTTTCGTACTGAAACATCTTAATCATGCACAGGAGACTTTCTAATGGCCAGCAGCGGCACCACCAGCACCCGCAAACGCTTCACGGGCGCCGAGTTCATCGTCCACTTCTTGGAGCAGCAGGGCATCAAGATCGTCACCGGCATCCCTGGCGGCAGCATCCTGCCGGTGTACGATGCCCTCAGCCAGAGCACCCAGATCCGCCACATCCTGGCTCGCCATGAACAAGGCGCGGGCTTCATCGCCCAGGGCATGGCCCGCACCGACGGCAAGCCCGCCGTCTGCATGGCGTCGAGCGGTCCGGGCGCCACCAATCTGGTCACCGCAATCGCCGACGCCCGTTTGGATAGCATCCCGCTGATCTGCATCACGGGCCAGGTGCCAGCCAGCATGATAGGCACCGATGCCTTCCAGGAGGTGGACACCTACGGCATCAGCATCCCCATCACCAAGCATAACTACTTGGTGCGCCACATCGAGGAACTCCCGCAGGTGATGTCCGATGCCTTCCGCATCGCCCAGTCGGGTCGGCCAGGCCCAGTTTGGATCGATATCCCGAAAGACGTCCAGACCGCCGTGTTCGAAATCGAAACCCAGCCCGCGATGGCTGAGAAAGCCGCGGCTCCGGCCTTCAGCGAAGAAAGCATCCGCGACGCCGCGGCTATGATCAACGCCGCAAAGCGCCCCGTGCTGTACCTGGGCGGCGGTGTCATCAATGCCCCAGCACGCGTGCGCGAACTGGCCGAGAAGGCCCAGCTTCCGACCACCATGACCCTTATGGCTCTGGGTATGCTGCCGAAGGCTCACCCGCTCTCGCTGGGTATGCTCGGGATGCACGGCGTCCGGAGCACCAACTACATCCTCCAGGAGGCCGACCTGCTGATCGTCCTGGGCGCCCGCTTCGACGACCGTGCCATCGGCAAAACCGAGCAGTTCTGCCCGAACGCCAAAATCATCCATGTTGACATTGACCGCGCGGAGTTGGGCAAGATCAAGCAGCCGCACGTGGCCATCCAGGCGGATGTGGACGACGTGCTGGCCCAGCTCATCCCGCTCGTGGAGGCACAGCCGCGCGCCGAATGGCACCAGCTGGTGGCGGACCTTCAACGCGAGTTCCCTTGCCCCATCCCCAAGGCCTGCGATCCCCTGAGCCATTACGGTCTGATCAACGCTGTGGCCGCGTGCGTCGATGACAACGCGATCATCACCACCGATGTGGGTCAACACCAGATGTGGACCGCTCAGGCGTACCCGCTGAACCGCCCGCGCCAGTGGCTCACCAGCGGCGGCCTGGGCACGATGGGGTTCGGTCTGCCCGCGGCCATCGGGGCTGCCCTGGCTAACCCAGACCGCAAGGTGCTGTGCTTCAGCGGTGACGGGAGCCTGATGATGAACATCCAGGAGATGGCCACCGCCAGCGAGAACCAGCTCGACGTCAAGATCATTCTGATGAACAACGAAGCCCTGGGCTTGGTACACCAGCAGCAGAGCCTGTTCTATGAACAGGGCGTCTTCGCCGCAACCTACCCCGGCAAGATTAACTTCATGCAGATCGCAGCCGGGTTCGGGCTGGAAACCTGCGATCTCAATAATGAGGCTGACCCGCAGGCGTCGCTCCAGGAAATCATCAACCGGCCCGGCCCGGCCCTGATCCATGTCCGTATCGACGCCGAGGAGAAGGTGTATCCAATGGTGCCCCCCGGCGCCGCCAACACGGAGATGGTCGGCGAGTGAAGTGATAGACTCAAGGTCGCTCCTAGCGAGTGGCCTTTATGATTATCACTTTAAATAAAAAAGGCACGTCAGATGACGTGCCTTTTTTCTTGTGCGGCCGC


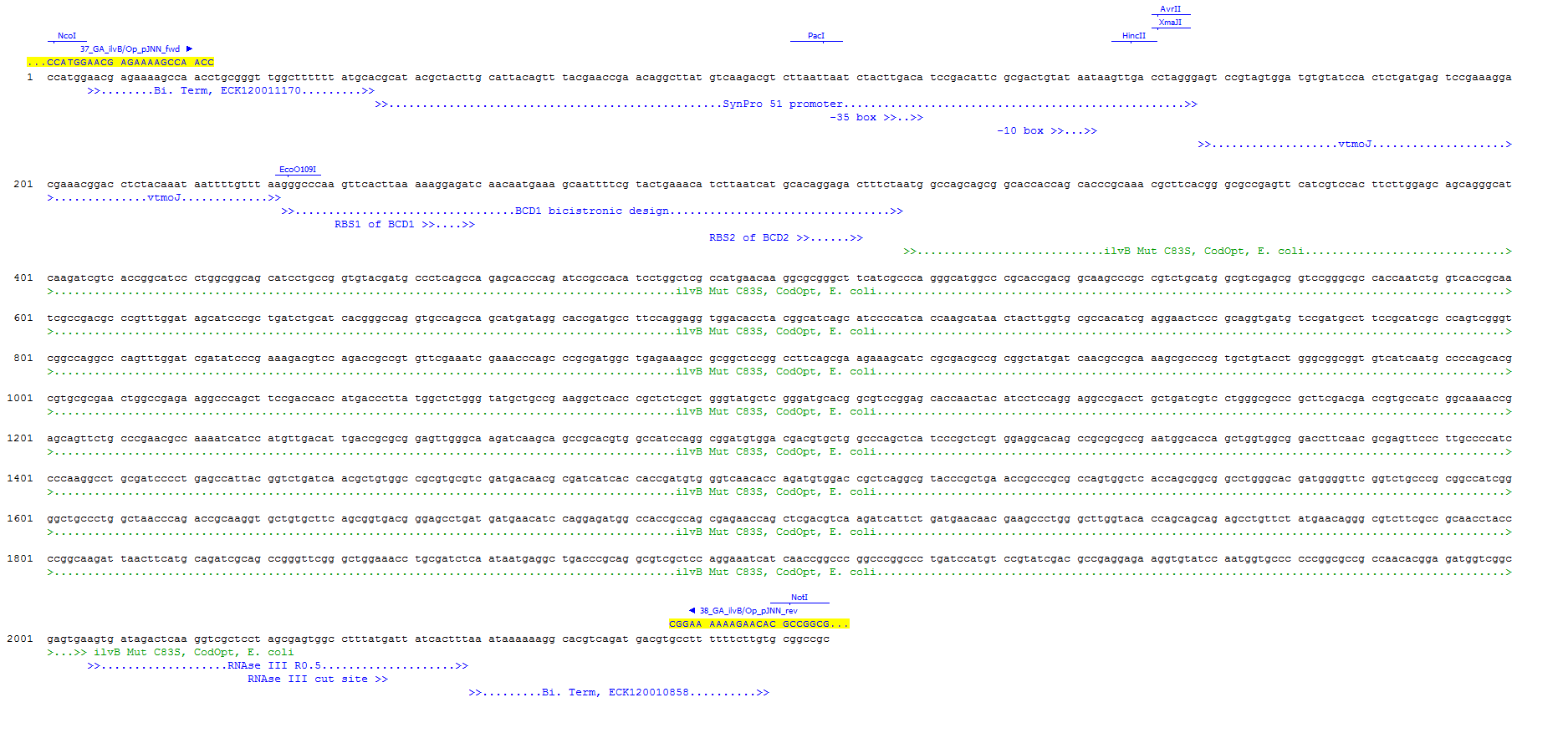


Supplementary Figure 2. Annotated sequence of the synthetic DNA fragment ordered, containing the bidirectional terminators, the synthetic Syn51 promoter, VtmoJ, BCD1, codon optimized *ilvB* C83S gene from *E. coli* and RNase III site R0.5.

Supplementary Figure 3. Linear regressions of standards of fluorescein in 0.1 mM borate buffer at pH 9.4 and respective fluorescence in arbitrary fluorescence units (AFU). measured with the m2p Biolector (488 nm/520 nm) at the gain 50 (violet), 60 (light blue), 70 (dark blue). The inset shows the linear regression equation and coefficient.

| Slope | -3.525 |
| --- | --- |
| Efficiency | 0.92171 |

Supplementary Figure 4. qPCR primer pair efficiency for the target gene *msfGFP*.

| Slope | -3.287 |
| --- | --- |
| Efficiency | 1.014785 |

Supplementary Figure 5. qPCR primer pair efficiency for the housekeeping gene *rpoB*.


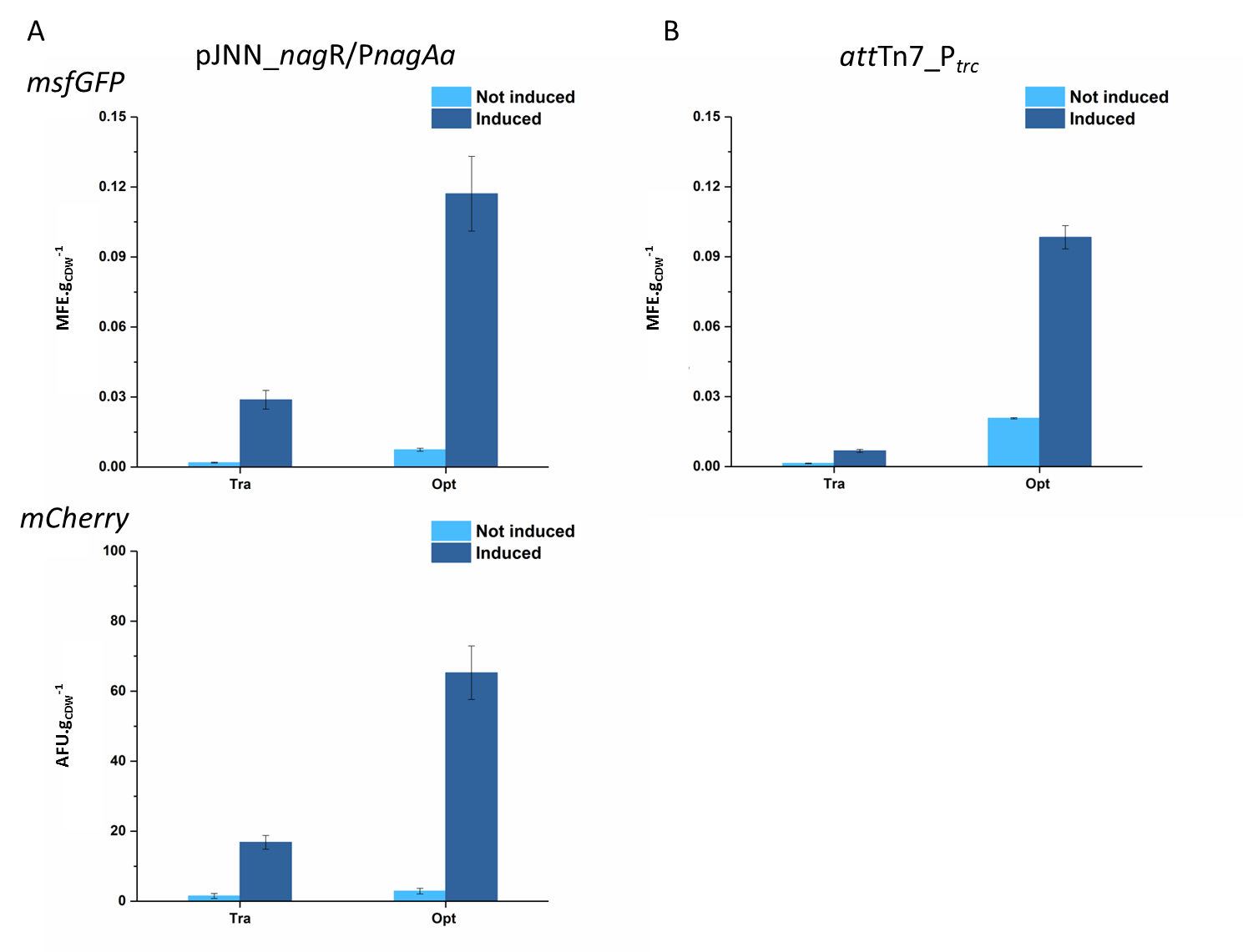


Supplementary Figure 6. Evaluation of the developed inducible gene expression constructs under induced (dark blue) and non-induced (light blue) conditions: (A) plasmid-based expression of *msfGFP* and *mCherry* under the control of the *nag*R/P*nagAa* promoter, (B) genomic integrated expression of *msfGFP* at the *att*Tn7 site under the control of the P*_trc_* promoter. Tra, traditional expression cassette; Opt, optimized expression cassette; MFE, µmoles of fluorescein equivalents; AFU, arbitrary fluorescence units.


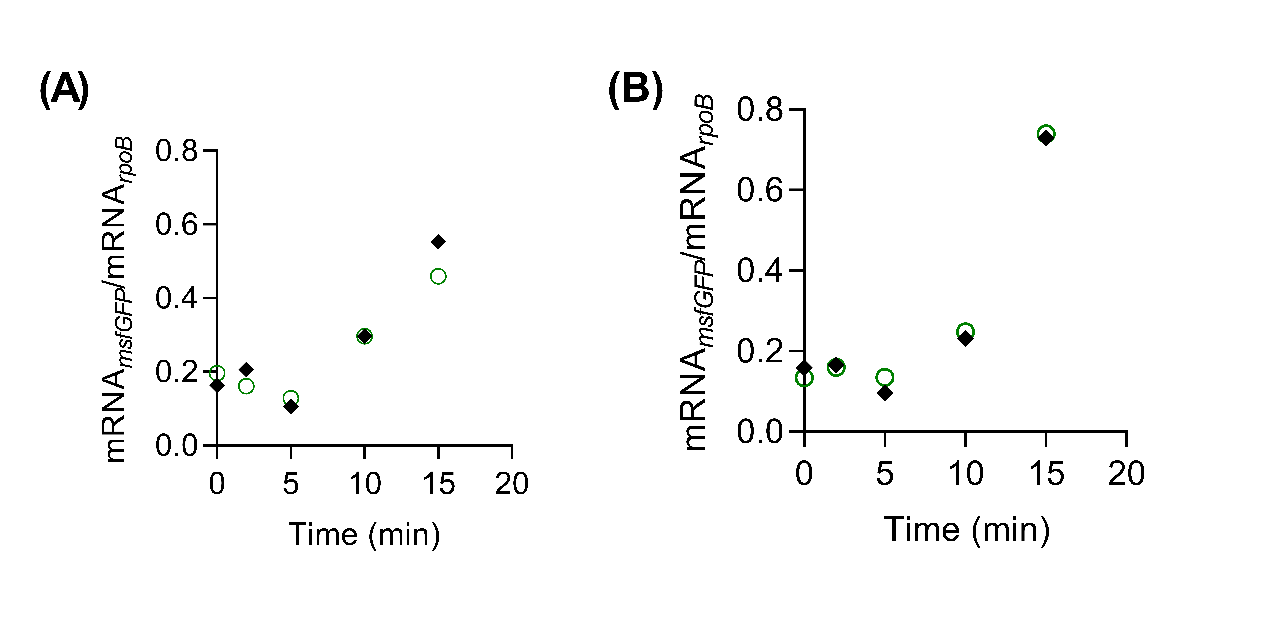


**Supplementary Figure 7.** Time course of mRNA abundance of the *msfGFP* gene normalized with the transcript level of the housekeeping gene *rpoB* for the traditional (A) and the optimized expression cassette (B).

**References**

Otto, M., Wynands, B., Drepper, T., Jaeger, K.-E., Thies, S., Loeschcke, A., et al. (2019). Targeting 16S ribosomal DNA for stable recombinant gene expression in *Pseudomonas*. *ACS Synth. Biol.*, acssynbio.9b00195. doi:10.1021/acssynbio.9b00195.
